# Comparison of the diagnostic significance of cerebrospinal fluid metagenomic next-generation sequencing copy number variation analysis and cytology in leptomeningeal malignancy

**DOI:** 10.1186/s12883-024-03655-7

**Published:** 2024-06-28

**Authors:** Le Zhang, Kechi Fang, Haitao Ren, Siyuan Fan, Jing Wang, Hongzhi Guan

**Affiliations:** 1 Department of Neurology, Peking Union Medical College Hospital, Chinese Academy of Medical Sciences and Peking Union Medical College, Beijing, 100730 China; 2 CAS Key Laboratory of Mental Health, Institute of Psychology, Chinese Academy of Sciences, Beijing, 100101 China; 3 Department of Psychology, University of Chinese Academy of Sciences, Beijing, 100049 China

**Keywords:** Copy number variations, mNGS, Cytology, Leptomeningeal malignancy diagnosis

## Abstract

**Background:**

Diagnosis and monitoring of leptomeningeal malignancy remain challenging, and are usually based on neurological, radiological, cerebrospinal fluid (CSF) and pathological findings. This study aimed to investigate the diagnostic performance of CSF metagenomic next-generation sequencing (mNGS) and chromosome copy number variations (CNVs) analysis in the detection of leptomeningeal malignancy.

**Methods:**

Of the 51 patients included in the study, 34 patients were diagnosed with leptomeningeal malignancies, and 17 patients were diagnosed with central nervous system (CNS) inflammatory diseases. The Sayk’s spontaneous cell sedimentation technique was employed for CSF cytology. And a well-designed approach utilizing the CSF mNGS-CNVs technique was explored for early diagnosis of leptomeningeal malignancy.

**Results:**

In the tumor group, 28 patients were positive for CSF cytology, and 24 patients were positive for CSF mNGS-CNVs. Sensitivity and specificity of CSF cytology were 82.35% (95% CI: 66.83-92.61%) and 94.12% (95% CI: 69.24-99.69%). In comparison, sensitivity and specificity of CSF mNGS-CNV were 70.59% (95% CI: 52.33-84.29%) and 100% (95% CI: 77.08-100%). There was no significant difference in diagnostic consistency between CSF cytology and mNGS-CNVs (*p* = 0.18, kappa = 0.650).

**Conclusions:**

CSF mNGS-CNVs tend to have higher specificity compared with traditional cytology and can be used as a complementary diagnostic method for patients with leptomeningeal malignancies.

## Background

Leptomeningeal malignancy is a severe condition associated with metastatic solid tumors and hematologic malignancies [1]. The most common tumors involving leptomeninges are lung cancer, breast cancer, lymphoma and leukemia and primary brain tumors such as gliomas, medulloblastomas, and ependymomas [1–3]. The presence of leptomeningeal malignancies is frequently associated with exceptional morbidity and mortality [4, 5]. However, diagnosing and effectively managing these conditions pose significant challenges.

Cerebrospinal fluid (CSF) analysis offers valuable diagnostic insights into leptomeningeal lesions. As a conventional morphological test, CSF cytology is instrumental in identifying tumor cells in individuals with leptomeningeal malignancy [6–8]. In recent years, metagenomic next-generation sequencing (mNGS) of CSF has emerged as a frontline diagnostic test for patients with meningitis with unknown etiology, enabling the identification of infectious pathogens. The majority of sequences obtained from mNGS correspond to human DNA, providing clinicians with an opportunity to utilize this wealth of sequencing information [9, 10].

Copy number variations (CNVs) refer to the deletion or amplification of DNA fragments at least 1 kb in length compared to the reference genome [11, 12]. Previous studies have highlighted the association between CNVs and tumor risk [13–15]. The diverse range of CNVs, in terms of their number and genomic distribution, reflects early genomic variations during tumorigenesis. Moreover, these variations may be linked to selection pressures on the tumor genome, representing distinct evolutionary processes and pathways within the tumor genome [16, 17].

Tumor cells release DNA fragments that circulate in CSF [18, 19]. Leveraging CSF sequencing analysis allows for more robust tumor diagnosis, particularly in cases with clinical suspicion but insufficient evidence. In this study, we employed mNGS-CNVs to investigate pathogenic microorganisms and structural variants within the human genome using CSF samples. Our aim was to explore an integrated diagnostic approach for leptomeningeal malignancy and CNS inflammatory diseases based on a single CSF mNGS-CNVs procedure. We assessed the utility of mNGS-CNVs in characterizing and monitoring leptomeningeal malignancy while comparing its performance with CSF cytology. The findings of this study provide valuable diagnostic references for implementing this technique in clinical practice.

## Methods

### Patient enrollment

**Fig. 1:**
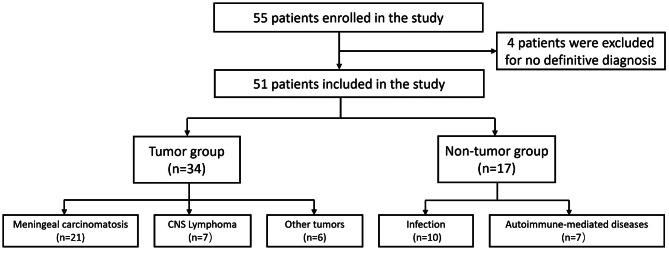
Patients in this study

In this prospective cohort study, we collected data from 55 patients in the Department of Neurology, Peking Union Medical College Hospital from March 2022 to February 2023. The inclusion criteria were as follows: (1) clinical or radiological manifestations of meningeal involvement; (2) diagnosis of leptomeningeal malignancy was suspected by clinicians. The exclusion criteria were as follows: (1) contraindications for lumbar puncture; (2) refuse CSF mNGS-CNV test; (3) no definitive diagnosis ultimately. Finally, 4 patients were excluded for no definitive diagnosis, and a total of 51 patients were included for further analysis (Fig. 1). Among these patients, 34 patients were diagnosed with leptomeningeal malignancies (tumor group), and 17 patients were diagnosed with CNS inflammatory diseases (non-tumor group).

### Diagnosis of CNS tumors

The diagnosis of CNS tumors was primarily based on the fifth edition of the WHO Classification of Tumors of the Central Nervous System (CNS) guidelines and the Chinese expert consensus on the diagnosis of meningeal carcinomatosis. To establish the diagnosis, the following criteria were considered: (1) Identification of malignant cells through pathological specimens obtained from surgery, biopsy, or CSF cytology; (2) Presence of typical neurological symptoms or signs of the disease, accompanied by MRI enhancement scans indicating the presence of lesions or meningeal enhancement; and (3) Reasonable exclusion of other diseases.

### CSF cytology

CSF samples for cytology were collected through lumbar puncture when patients presented with neurological symptoms and leptomeningeal malignancies were suspected. For patients with high suspicion of leptomeningeal malignancies but negative CSF cytology, some underwent lumbar puncture again for cytology test with informed consent. The Sayk’s spontaneous cell sedimentation technique was employed for further analysis. Briefly, 0.5 mL of CSF was placed into the spontaneous sedimentation chamber and stored overnight at 4 °C. The cells precipitated onto the slides. Then, the slides were air-dried and stained with May–Grunwald–Giemsa staining [7]. The morphological characteristics of tumor cells in CSF cytology were assessed, including cell morphology, cytoplasmic features, nucleus characteristics, presence of pigment granules or vacuoles in the cytoplasm, regularly or clustered arrangement of cells, and distinct morphological variations observed in tumor cells from different sources.

### Procedure and analysis of mNGS-CNVs

CSF samples for mNGS-CNVs detection were collected from the first lumbar puncture. The collected samples were centrifuged at room temperature, and then stored in the − 80 °C refrigerator. DNA was extracted from CSF using commercially available automated nucleic acid extraction kits. Following the extraction, library preparation was conducted using end-repair, ligation adaptor and other necessary procedures. Subsequently, CSF mNGS DNA sequencing was performed on the Illumina Nextseq platform (Illumina Nextseq CN550) at the V-Medical laboratory in China. To ensure the accuracy of the sequencing data, a rigorous quality control process was implemented.

The obtained sequencing reads were aligned to the human reference genome (hg19) using BWA-MeM software (version: 0.7.17-r1188) to identify both human and nonhuman sequences present in the samples. The nonhuman sequences were subjected to analysis using MiTopia-PAI, a self-built metagenomic data analysis software. This analysis aimed to identify pathogenic microorganisms and recognize clinical pathogenic microorganisms with high confidence.

For CNV analysis, the human sequences within the samples were analyzed using CNVkit (version: 0.9.9). To establish the baseline for CNV analysis, a dataset of tumor-negative samples was used. These baseline samples consisted of CSF samples obtained from patients diagnosed with autoimmune encephalitis. The results of the CNV analysis were visualized by setting the window size and applying a filtering threshold [20]. We determined the possibility of tumor when large CNVs (> 10 Mb) were detected.

### Statistical analysis

SPSS 29 (Statistical Package for the Social Sciences, version 29.0, IBM, New York, USA) was applied to the statistical analysis of the data. Data were expressed as proportions for categorical variables. Sensitivity and specificity, along with their corresponding 95% confidence intervals (CIs) of mNGS-CNVs were calculated. McNemar test was performed for statistical analysis. *P* < 0.05 was used to indicate statistical significance.

## Results

### Basic information

In total, 51 patients were included in the study (Fig. 1). The baseline characteristics are shown in Table 1. Tumor types in the study were as follows: leptomeningeal metastases of lung cancer (*n* = 10); leptomeningeal metastases of breast cancer (*n* = 6); leptomeningeal metastases of leukemia (*n* = 1); leptomeningeal metastases of gastrointestinal tumors (*n* = 1); B-cell lymphoma (*n* = 6); T-cell lymphoma (*n* = 1); diffuse midline glioma (*n* = 1); pleomorphic xanthoma astrocytoma(*n* = 1); rhabdomyosarcoma (*n* = 1); primitive neuroectodermal tumor (*n* = 2); spinal tumor (*n* = 1); primary tumor unknown (*n* = 3).

**Table 1:** Clinical manifestations and auxiliary examinations of patients

Number (%)	Tumor group (*n* = 34)	Non-tumor group (*n* = 17)
Characteristic		
Age (y); median (range)	47 (10–79)	57 (2–76)
Sex		
Male	14 (41.18%)	9 (52.94%)
Female	20 (58.82%)	8 (47.06%)
Clinical manifestations		
Headache	18 (52.94%)	9 (52.94%)
Fever	0	7 (41.18%)
Diminution of vision	3 (8.82%)	2 (11.76%)
Cognitive impairment	5 (14.71%)	1 (5.89%)
Consciousness disorder	5 (14.71%)	5 (29.41%)
Limb numbness	3 (8.82%)	2 (11.76%)
Limb weakness	5 (14.71%)	2 (11.76%)
Diplopia	4 (11.76%)	3 (17.65%)
CSF characteristics		
Increased pressure	7 (20.59%)	6 (35.29%)
Increased protein	22 (64.71%)	13 (76.47%)
Radiographic features (MRI)	*n* = 20	*n* = 7
Enhanced lesions	19 (95.00%)	6 (85.71%)

CSF pressure (> 180 mmH_2_O) was increased in 20.59% of the tumor group and 35.29% of the nontumor group. Increased total protein was present in 64.71% of the tumor group and 76.47% of the non-tumor group. In the tumor group, 20 patients completed enhanced MRI examinations, with 19 (95.00%) patients displaying enhanced lesions or meningeal involvement. In the non-tumor group, 7 patients completed MRI, and 6 (85.71%) patients had abnormal signals.

### CSF cytology and CNVs analysis

**Fig. 2:**
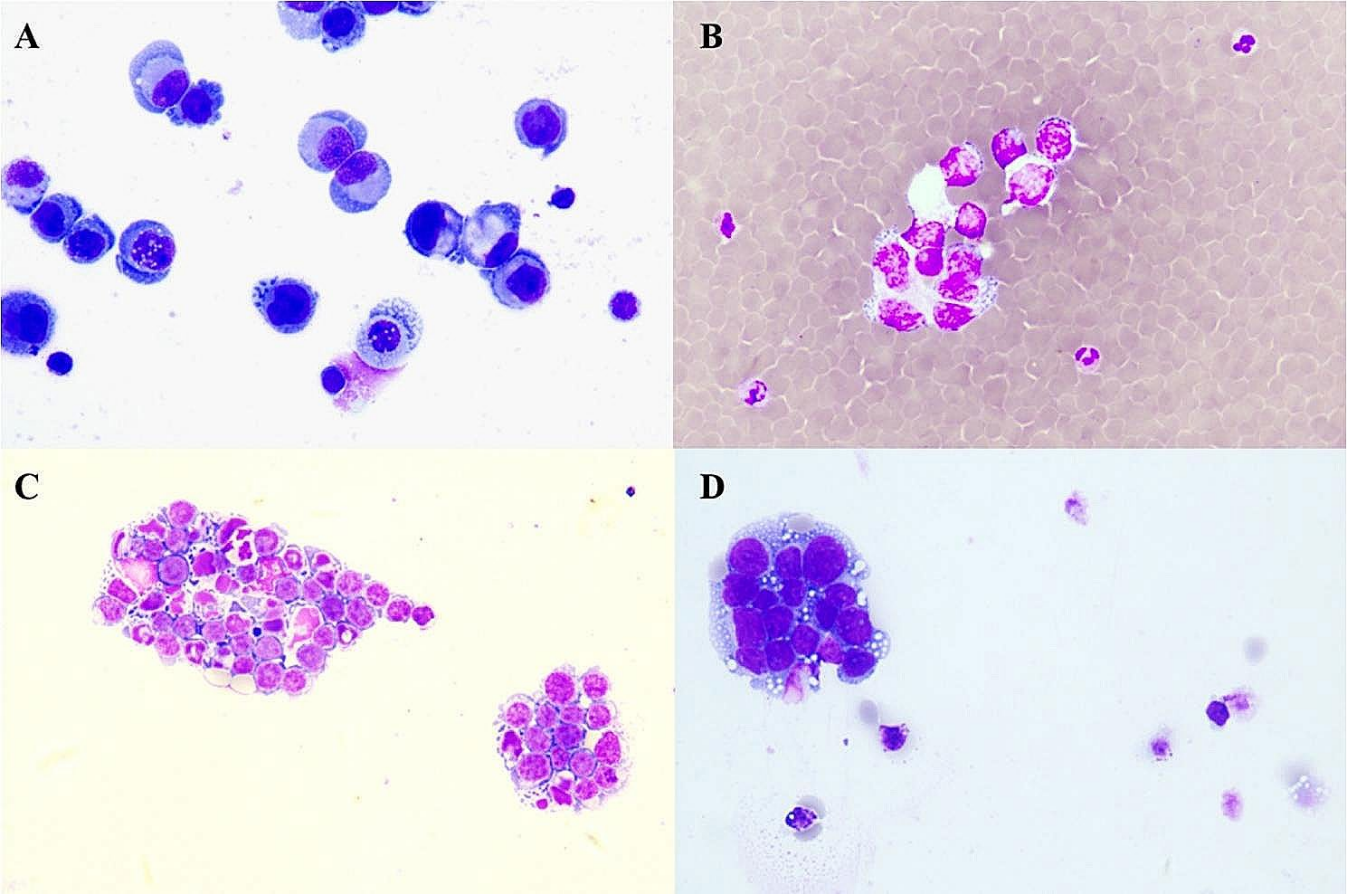
CSF cytology of leptomeningeal malignancies. CSF cytology of tumor cells (spontaneous cell sedimentation technique, May-Gruwald-Giemsa staining ×200). (**A**) Patient 8: leptomeningeal metastases of breast cancer. (**B**) Patient 23: diffuse midline glioma. (**C**) Patient 30: CNS B-cell lymphoma. (**D**) Patient 34: rhabdomyosarcoma

**Fig. 3:**
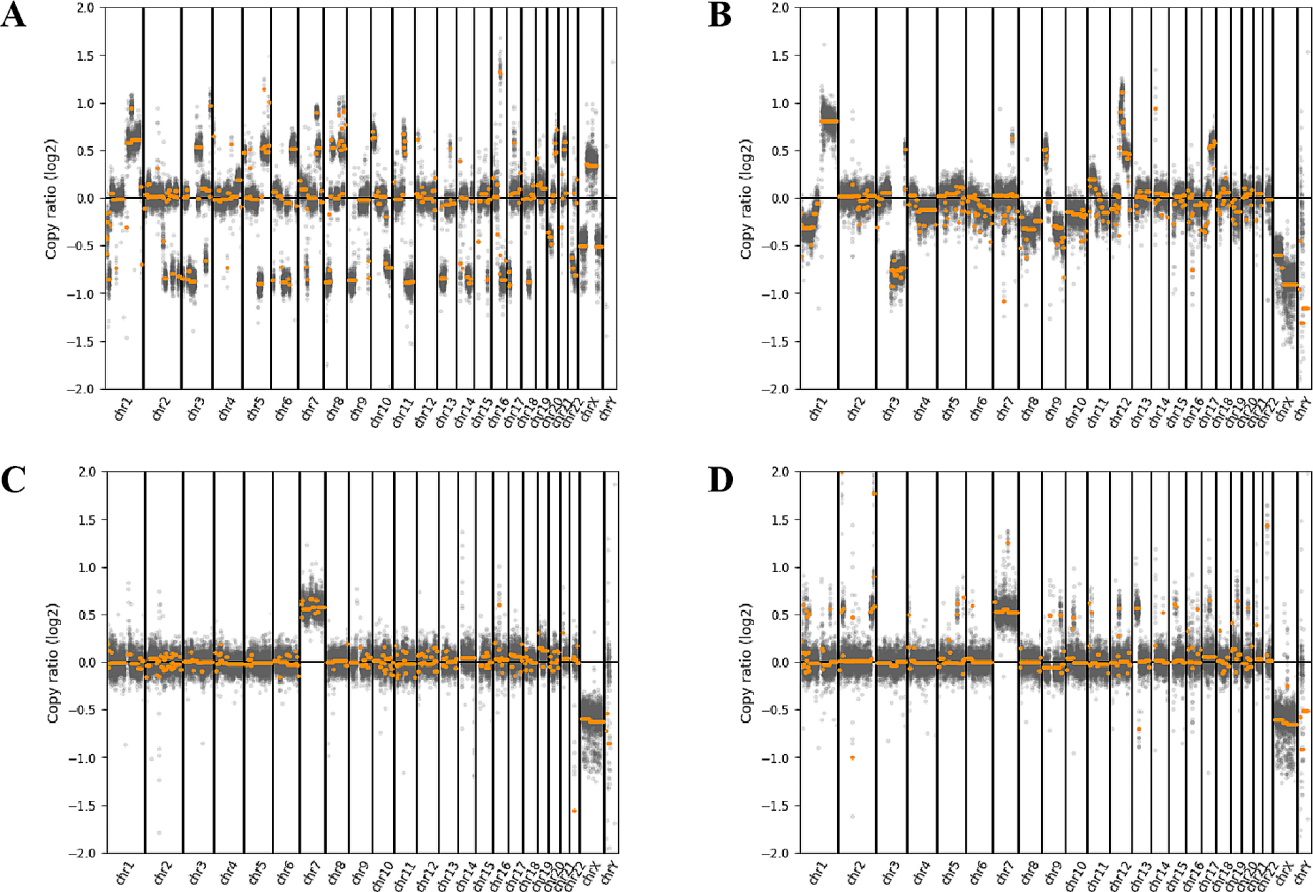
CSF CNV analysis in patients with leptomeningeal malignancies. CNV segments in patients with leptomeningeal malignancies. (**A**) Patient 8: leptomeningeal metastases of breast cancer, 32 CNV segments. (**B**) Patient 23: diffuse midline glioma, 12 CNV segments. (**C**) Patient 30: CNS B-cell lymphoma, 5 CNV segments. (**D**) Patient 34: rhabdomyosarcoma, 5 CNV segments

In the tumor group, 28 (82.35%) patients had positive CSF cytology, and representative positive results are presented in Fig. 2. Among the 6 (17.65%) patients with negative cytology, one patient was diagnosed with meningeal carcinomatosis (MC), 3 patients with CNS lymphoma, and 1 patient with a primitive neuroectodermal tumor. Additionally, 24 (70.59%) patients had positive CSF CNV results, and some examples are presented in Fig. 3. The median CNV segment size was 10.5 (range: 1 ~ 49). No CNV segments larger than 10 Mb were detected in the remaining 10 (29.41%) patients with negative CNV results. Among those with negative CNV results, 3 patients were diagnosed with MC, 6 patients with CNS lymphoma, and 1 patient with glioma (Table 2).

In the non-tumor group, the CSF cytology of 16 (94.12%) patients showed inflammatory changes, and the cytology of 1 (5.89%) patient reported atypical-abnormal cells, which were considered tumor cells. All 17 patients exhibited negative CSF CNV results.

**Table 2:** Comparison of CSF cytology and mNGS-CNVs.

Leptomeningeal Malignancies	Cytology		mNGS-CNVs	
	Positive	Negative	Positive	Negative
leptomeningeal metastases of lung cancer	9	1	9	1
leptomeningeal metastases of breast cancer	6	0	5	1
leptomeningeal metastases of leukemia	1	0	1	0
leptomeningeal metastases of gastrointestinal tumors	1	0	1	0
B-cell lymphoma	4	2	1	5
T-cell lymphoma	0	1	0	1
Diffuse midline glioma	1	0	1	0
Pleomorphic xanthoma astrocytoma	0	1	0	1
Rhabdomyosarcoma	1	0	1	0
Primitive neuroectodermal tumor	1	1	2	0
Spinal tumor	1	0	1	0
Primary tumor unknown	3	0	2	1
Total	28	6	24	10

### Sensitivity and specificity

The results demonstrated that for the diagnosis of leptomeningeal malignancy, CSF cytology exhibited a sensitivity of 82.35% (95% CI: 66.83-92.61%) and specificity of 94.12% (95% CI: 69.24-99.69%). Similarly, CSF mNGS-CNVs displayed a sensitivity of 70.59% (95% CI: 52.33-84.29%) and a specificity of 100% (95% CI: 77.08-100%). Moreover, two patients exhibited CNV segments larger than 3 Mb but smaller than 10 Mb. Adjusting the CNV length reference to 3 Mb resulted in a sensitivity of 76.47% (95% CI: 58.43-88.62%) for CSF mNGS-CNVs. Importantly, no significant difference in diagnostic consistency was observed between CSF cytology and mNGS-CNVs (*p* = 0.18, kappa = 0.650).

## Discussion

In this study, we applied CSF mNGS-CNVs analysis and cytological examinations in a cohort of 51 patients diagnosed with leptomeningeal malignancies and CNS inflammatory diseases. The main findings are summarized as follows. Firstly, we established a well-designed approach for the analysis of the CSF mNGS-CNV technique, which detected both neoplasms and pathogenic microorganisms and provided results in hours to facilitate the earlier diagnosis of leptomeningeal malignancies. Secondly, there was no significant difference of diagnostic consistency between CSF cytology and mNGS-CNVs, which suggested that mNGS-CNVs may be considered as an auxiliary examination in hospitals that do not have the conditions to undertake CSF cytological testing. Lastly, mNGS-CNVs does not require intact CSF cells and can provide highly specific results without the need for repeated lumbar puncture.

The timely diagnosis of leptomeningeal malignancy remains challenging, often requiring prompt CSF cytology, tumor tissue pathology, gene mutation detection, and other auxiliary examinations [21–23]. While cytology is a classical method in the diagnosis of leptomeningeal malignancy, its diagnostic accuracy may vary and heavily relies on experienced cytopathologists [22–24]. Moreover, the positive rate of cytology from a single lumbar puncture has its limitations. In certain centers, standardized procedures for CSF cytology may not be uniformly implemented. Therefore, to explore more suggestive diagnostic techniques that offer greater diagnostic accuracy for leptomeningeal malignancies is meaningful.

Previous studies have demonstrated that gene profiles in CSF can provide valuable insights into CNS tumors [19, 25, 26]. Recently, researchers have found that mNGS of human peripheral blood, bronchoalveolar lavage fluid, CSF or other body fluids provide clues on distinguishing malignancies [27–29] from infectious/inflammatory diseases [30–32]. This approach was mainly based on CNV analysis, which exhibits relatively high specificity. Gu et al. [10] reported that the mNGS-CNVs had a sensitivity of 71% and a specificity of 100% in detecting CNS malignant tumors by using the earliest CSF specimens. Fifty-five patients of CNS malignant neoplasms were included in the study, while 65.5% of them were diagnosed with lymphomas, which may not seem to be comprehensive enough to cover various neoplasms’ CNVs and cytological characteristics.

In our study, moderate sensitivity and high specificity of the CSF mNGS-CNVs technique compared with cytology provide evidence for its feasibility and potential as an auxiliary method in the diagnosis of leptomeningeal malignancies. Additionally, CSF mNGS testing contains information of suspicious microorganisms for differential diagnosis of infectious encephalitis or meningitis. In addition, mNGS-CNVs analysis require smaller CSF volume and do not rely on the integrity of cells and repeated lumber punctures.

However, CNV analysis can be affected by some confounding factors, such as the complexity of tumor samples and the detection thresholds. Challenges from the complex tumor samples including tumor types, the aneuploidy of the genome and tumor fraction [33]. Different tumor types have different CNV patterns [34, 35]. For example, the number of CNV was higher in aggressive tumors than indolent ones [36]. We noticed that large CNVs of CNS lymphoma was rarely detected in our study. Given this perspective, we speculated that these tumors may harbor lower fractions of circulating tumor DNA or less whole genome doubling events. Besides, CNS lymphoma is difficult to diagnose relying on single auxiliary test [23], and reasonable combination of CSF cytology, CNVs, flow cytometry and other diagnostic tests to establish the diagnosis is recommended.

On the other hand, there is no consensus on the setting of criteria for CNV detection threshold. In our study, the sensitivity of CSF mNGS-CNVs was 70.59% when using 10 Mb as large CNVs threshold, which is similar to Gu et al. [10] with the same threshold. In previous studies, region of 3 Mb in the human genome contains one gene or only a few genes, and may be detected in the tumor genetic variations [37]. When adjusting the cutoff value to 3 Mb, the diagnostic sensitivity of mNGS-CNVs increased to 76.47% in this study. This phenomenon may have some implications for future research.

## Conclusions

Accurate detection of CNVs in the human genome and subsequent analysis of the biological implications of these variations hold significant importance for tumor diagnosis. Overall, our study employed a well-designed and comprehensive approach to analyze CSF samples, which can contribute to the differential diagnosis of CNS malignancies and infectious diseases. Nevertheless, further research with larger sample sizes is warranted to validate and expand upon these findings, ensuring a more robust understanding of the diagnostic capabilities of mNGS-CNVs analysis in various types of CNS tumors.

## Data Availability

The datasets generated during the current study has been uploaded to CNCB database, https://www.cncb.ac.cn/ (Accession: PRJCA023198).
